# Ordered perovskite nanocrystals: a transformative platform for optoelectronic applications

**DOI:** 10.1039/d5sc08666c

**Published:** 2026-02-09

**Authors:** Lujun Zhai, Huifeng Li, Tom Wu, Jianyu Yuan

**Affiliations:** a State Key Laboratory of Bioinspired Interfacial Materials Science, Institute of Functional Nano & Soft Materials (FUNSOM), Soochow University 199 Ren-Ai Road, Suzhou Industrial Park Suzhou Jiangsu 215123 China jyyuan@suda.edu.cn; b Department of Applied Physics Hong Kong Polytechnic University Hong Kong Hong Kong SAR 999077 China tom-tao.wu@polyu.edu.hk

## Abstract

Perovskite nanocrystals (PNCs) have emerged as a versatile platform for next-generation optoelectronics owing to high photoluminescence quantum yields, tunable bandgaps, and superior charge transport. Yet, the intrinsic disorder of colloidal systems and limitations of scalable processing severely restrict their performance. The structurally ordered PNCs, called herein as OPNCs, has emerged as a promising strategy to overcome the intrinsic limitations of disordered colloidal systems. Controllable self-assembly enables the formation of ordered superlattices, where collective effects such as enhanced carrier mobility, improved photoluminescence, and miniband formation can be realized. In this perspective, we highlight recent advances in solvent engineering, functionalized ligand design, and external-field modulation that provide new levers for achieving structural control. We further discuss how ordered architectures open pathways toward device applications such as pixelated light-emitting devices, low-threshold lasers, and polarization-sensitive photodetectors. By reframing self-assembly as a controllable and designable process, we propose that OPNC superlattices hold transformative potential for stable and high-performance optoelectronic applications.

## Abstract 2

Perovskite nanocrystals (PNCs) have emerged as a versatile platform for next-generation optoelectronics owing to high photoluminescence quantum yields, tunable bandgaps, and superior charge transport.

## Graphical abstract

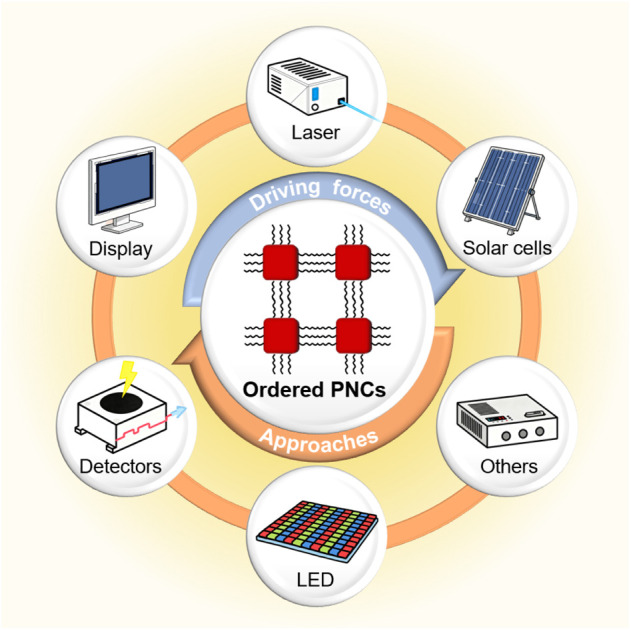

## 1. Introduction

Emerging perovskite nanocrystals (PNCs) show high photoluminescence quantum yields, tunable bandgaps, and solution processablity, making them attractive candidates for next-generation optoelectronics.^1–18^ However, despite these intrinsic advantages, the fully application of PNCs requires overcoming several critical challenges.^19^ In particular, the morphological homogeneity of individual nanocrystals,^20–22^ the environmental stability of PNCs,^22–25^ the defect control and passivation of nanocrystals^21,25,26^ and the charge transport of nanocrystals^27,28^ are some of the main critical issues determining the performance of PNC based devices.

In most practical applications, the deposition of nanocrystals as functional layers typically relies on solution-processable techniques such as spin coating,^29^ melt extrusion,^30^ spray drying,^31^ inkjet printing,^32^ and blade coating,^1,33^*etc.* These methods, while scalable, inherently introduce a certain degree of disorder. Macroscopically, it is shown as the roughness of the surface and interior of the nanocrystal film,^22,34–36^ and the decline of the optoelectronic performance. This disorder stems from two primary sources: (i) intrinsic structural and surface-related imperfections within the nanocrystals, including lattice defects, ligand desorption, size heterogeneity, and interparticle interactions; and (ii) the mismatch between the nanoscale dimensions of the nanocrystals and the microscale resolution of conventional processing techniques, which limits spatial control during deposition. Disorder at the nanoscale often leads to poor electronic coupling, increased trap densities, and inhomogeneous charge transport pathways, all of which critically undermine device efficiency and operational stability.

In recent years, the realization of ordered stacking architectures has emerged as a promising strategy to overcome the intrinsic performance limitations associated with randomly disordered stacking^37,38^ and aging of the ordered systems.^39^ To address these disordering challenges, researchers have developed a variety of approaches to improve the ordering of PNCs. These methods aim to simultaneously enhance the crystallinity, size uniformity, and spatial organization of PNCs, thereby promoting coherent collective behaviors such as enhanced carrier mobility,^40^ improved photoluminescence,^41^ and miniband formation.^34,42^ Recent efforts have focused on structural engineering of PNC assemblies. In particular, the creation of ordered stacking architectures has emerged as a powerful route to unlock collective properties beyond those of individual nanocrystals. At a large scale compared to NCs dimensions, typically greater than or equal to tens of micrometers, ordered and densely stacked structure formed over a large area by the PNCs nanocrystals which the degree of order support the formation of the wavefunction-coupling and the improvement of the carrier transportation defined as OPNC structures. Among these, nanocrystal superlattices and long-range ordered layered films can both be referred to as OPNC structures. This perspective explores the driving forces and strategies for controllable self-assembly and highlights the opportunities such ordered structures create for high-performance optoelectronic devices.

## 2. Mechanisms and prospects for controllable self-assembly

**Fig. 1:**
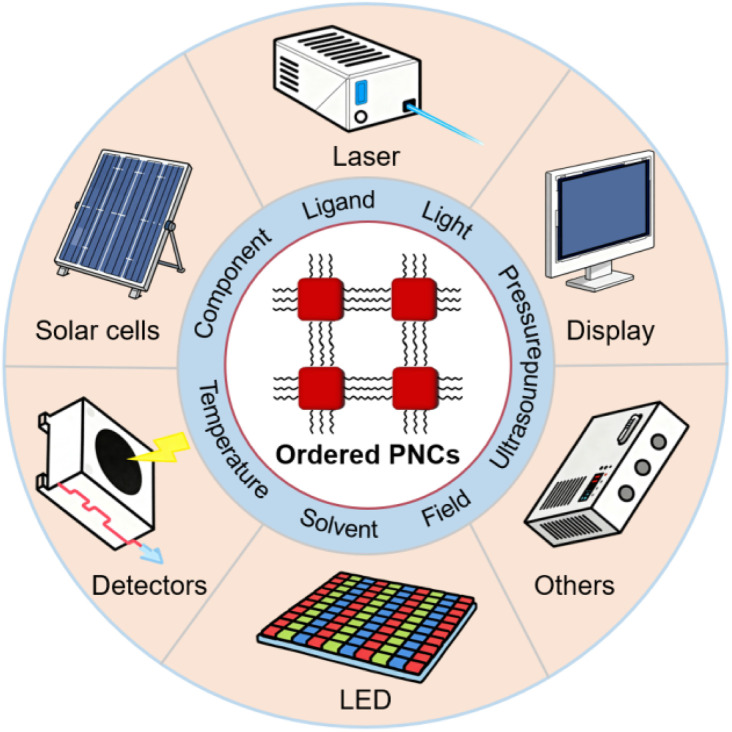
Schematic diagram of PNC self-assembly methods and emerging applications.

Self-assembly is a ubiquitous phenomenon in nature, is exemplified by the formation of iconic structures such as the DNA double helix.^43^ In this process, molecules or nanoscale components spontaneously organize into structured, functional arrangements, *e.g.*, complex supramolecular architectures, under microscopic forces, such as van der Waals interactions, hydrogen bonding, or electrostatics. Broadly speaking, the assembly of PNCs constitutes a multi-scale, hierarchical self-assembly process, spanning from the molecular precursor organization and nanocrystal formation to the spatial ordering of the PNCs themselves, and finally to the emergence of ordered micrometer- or millimeter-scale architectures. In the broad family of quantum dots (QDs), different material systems exhibit distinct morphologies, crystal types, luminescence mechanisms, and optoelectronic properties. PNCs possess a cubic morphology that differs from the spherical shape to most QDs, and they belong to the unique class of ionic crystals. This characteristic renders the formation of ordered structures considerably more complex compared to spherical QDs. Furthermore, their distinctive cubic morphology endows them with macroscopic anisotropy, which holds significant research and application value in both electrical and optical fields. Moreover, researchers have developed various methods to induce the self-assembly of PNCs, and external factors such as solvent, ligand, and external fields have been leveraged to control the ordering morphologies, which in turn are expected to impact the advancement of a plethora of optoelectronic applications (Fig. 1).

### 2.1 The driving forces and influencing factors of self-assembly

The process of self-assembly spans a wide range of length scales. At each length scale, distinct driving forces and physicochemical factors govern the motion and assembly of basic units. Self-assembly is a process that transitions a system from a disordered to an ordered state. Regardless of the intermediate stages, it is inherently driven by entropy and enthalpy, and the final system represents a thermodynamically stable outcome of entropy–enthalpy balance.^44^ The driving forces include intrinsic interactions such as random Brownian motion, van der Waals attraction,^45^ hydrogen bonding,^46^ ligand interactions,^47^ and morphology-dependent effects,^21^ as well as extrinsic influences such as forces of fields (electric force,^48,49^ acoustic force,^34^ electromagnetic field^50^*etc.*), wettability,^51,52^ temperature,^53^ and confinement imposed by templates.^51,52^ In the initial stage of self-assembly, PNCs are individually dispersed in the solvent (normally hexane, toluene, octane), where random Brownian motion predominates. As the solvent evaporates, the PNCs begin to approach one another. When they come within a certain proximity, electromagnetic interactions between them drive an orderly aggregation into a certain, often cubic, geometric structure. The dynamic balance between these cooperative and competing factors gives rise to the complexity of nanocrystal assembly. In general, the micro and macro factors have also provided researchers with a rich variety of strategies to achieve controllable PNC self-assembly. Currently, the research is still in its infancy, and the physical and chemical methods employed are relatively limited. Various physical and chemical factors influencing nanocrystal self-assembly have not been comprehensively utilized, which, on the other hand, also offers unlimited possibilities and opportunities for future explorations.

### 2.2 Prospects for controllable self-assembly

Based on the environment in which the process occurs, self-assembly can be categorized into solution-mediated assembly and substrate-mediated assembly. Solution-mediated assembly primarily relies on external fields and internal microscopic force to manipulate the behavior and movement of PNCs in the solvent, whereas substrate-mediated assembly depends more on microscopic forces and templating confinement to achieve controlled organization. In the past few decades, researchers have made considerable progress in the controlled self-assembly^54^ of traditional non-perovskite nanocrystals,^51,55^ metal-based^48,56^ and other microparticles.^57^ These findings offer valuable foundations for the controllable self-assembly of emerging PNCs. However, it is important to note that ionic PNCs exhibit unique intrinsic characteristics. Primarily, their crystalline lattice possesses a face-centered cubic structure, which directly dictates the cubic morphology of individual PNC. In comparison with spherical nanoparticles, such anisotropic cubes possess significantly fewer rotational degrees of freedom during the assembly process. This inherent geometric constraint consequently makes the precise control and manipulation of orientation within PNCs superlattices considerably more challenging.

#### 2.2.1 Solvent-driven self-assembly

**Fig. 2:**
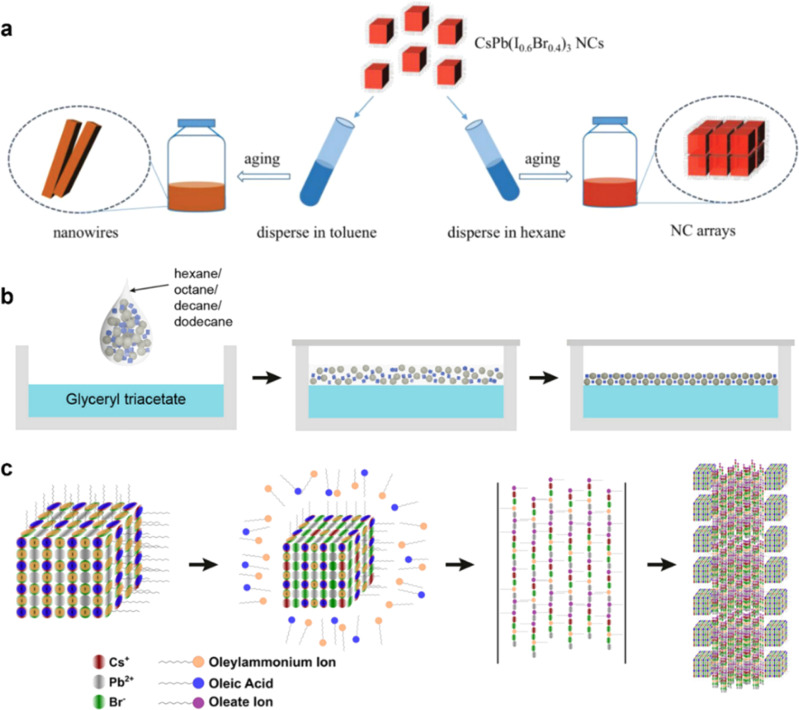
(a) Illustration of the morphology evolution of CsPb(I_0.6_Br_0.4_)_3_ PNCs in toluene and hexane solvent.^59^ Copyright 2022, Wiley-VCH. (b) Self-assembly of perovskite NCs at the liquid–air interface.^63^ Copyright 2022, American Chemical Society. (c) Schematic describing the mechanism behind CsPbBr_3_ PNC self-assembly into 1D superlattice chains.^58^ Copyright 2017, American Chemical Society.

For solution-mediated assembly, there have been some reports which succeeded in utilizing various external fields and internal force to guide the organization of nanostructures,^34,50,58,59^ In solution, the solvent emerges not merely as a passive medium, but as a critically active component which can be leveraged to the structural outcomes (Fig. 2a). As is well-established, PNCs is an ionic crystal wrapped with all kinds of cap ligands whose inherent ionic nature dictates that the polarity of external solutions or environments plays a critical role in the stability and growth of perovskite crystals and the adhesion of the ligands. Under high-polarity conditions, such as in ethanol solutions, the high polarity environment will disrupt the ligand adhesion and the PNC tend to dissolve, Consequently, in the process of solvent-mediated ordered assembly of nanocrystals, the limit of polarity that different perovskite nanocrystals can tolerate has not been explored and clearly defined. This remains further research. Existing studies have demonstrated that solvent polarity exerts profound influence over the morphology, orientation, and even the kinetic pathways of self-assembled structures.^60,61^ This sensitivity to the dielectric environment—affecting colloidal stability, interaction potentials, and ligand conformation—suggests an underutilized opportunity: temporally resolved solvent engineering. Rather than maintaining static solvent conditions, the modulation of polarity during different stages of assembly presents a promising paradigm for achieving control over superstructure characteristics. This strategy represents a significant and promising endeavor for achieving control over the orientation and morphology in self-assembly. In addition to common polar and non-polar solvents, certain chemical solvents from other systems (such as mineral oil^62^) are also expected to play a role in the self-assembly process (Fig. 2b). Chemical reactions occurring at the interface between two systems and the differences in surface tension resulting from distinct systems will influence the self-assembly of nanocrystals (Fig. 2c).^63^ A common method utilizing this principle is the formation of Langmuir–Blodgett films.^64^ These hold considerable importance for enhancing the understanding of the sensitivity of each assembly stage to polarity and environmental conditions, as well as for leveraging solvent properties to facilitate precisely controlled self-assembly processes.

#### 2.2.2 Functional ligand-driven self-assembly

**Fig. 3:**
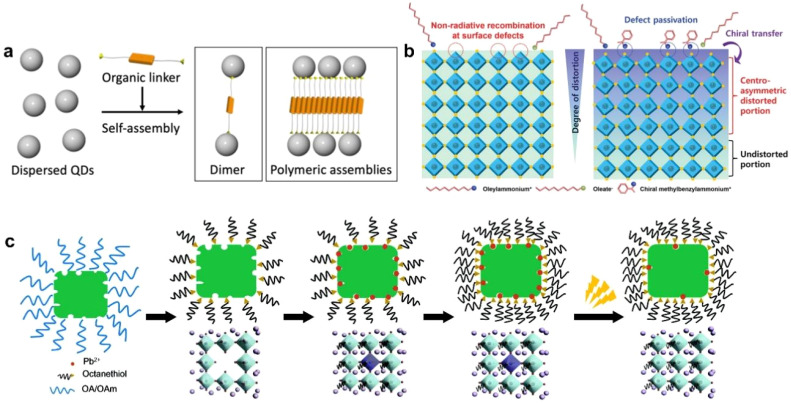
(a) Schematic of the self-assembly of QDs into polymeric assemblies with organic linkers.^65^ Copyright 2020, Chemistry Europe. (b) Schematic illustrations of colloidal PNCs without chiral ligands and chiral transfer mechanism in surface-functionalized colloidal PNCs with chiral ligands.^66^ Copyright 2022, Wiley-VCH. (c) Schematic of the surface treatment and photoactivation process.^67^ Copyright 2017, Royal Society of Chemistry.

When nanocrystals are close to each other, the surface ligands play an important role in regulating the assembly of nanocrystals.^59,65^ Ligand engineering in PNCs has evolved from passive stabilization to active functional encoding, enabling the design of superlattices with tailored properties. Conductive ligands such as thiocyanates, conjugated molecules (Fig. 3a) enhance interparticle electronic coupling, facilitating efficient charge transport for optoelectronic applications. Chiral ligands (Fig. 3b) induce circularly polarized luminescence by transferring asymmetry to the superlattice, offering potential in quantum optics and sensing. Environment-responsive ligands (*e.g.*, thermotropic polymers) enable dynamic structural reconfiguration under external stimuli (light, temperature) (Fig. 3c), paving the way for adaptive meta-materials. By rationally designing ligand structure, PNC superstructures can be programmed at the molecular level to integrate multiple functionalities—such as conductivity, chirality, and stimuli-responsiveness—into superstructures. This paradigm shift positions ligands as key determinants of superstructures functionality, bridging nanoscale assembly to macroscopic device performance. Currently, research on manipulating the properties of perovskite nanostructures through the design of functionalized ligands has primarily focused on disordered PNC systems.^66–68^ Reports on introducing such ligands into superlattices and exploring their resulting effects and characterization remain scarce. In ordered superlattice systems, ligands or connectors, as crucial components, are anticipated to play more significant roles and exhibit unique properties worthy of in-depth investigation.

#### 2.2.3 External modulation-driven self-assembly

**Fig. 4:**
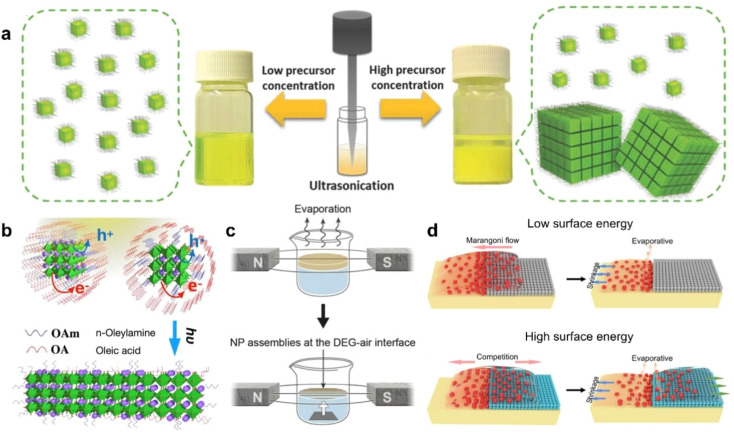
(a) Schematic diagram of the synthesis and self-assembly of CsPbBr_3_ NCs by single-step tip sonication of precursor salts in octadecene.^34^ Copyright 2018, Wiley-VCH. (b) Illustration of light-induced ligand removal mechanism.^50^ Copyright 2019, American Chemical Society. (c) Self-assembly of magnetite nanocubes under the magnetic field.^48^ Copyright 2014, The American Association for the Advancement of Science. (d) Schematic showing the assembly principle of quantum dots with different interfaces of microstructures.^51^ Copyright 2018, Wiley-VCH.

In addition to the interactions among components within dispersion, external factors can also exert strong manipulative and controlling effects on the assembly of PNCs. As mentioned before, these external factors primarily include external forces such as electric fields, magnetic fields, optical fields, acoustic fields, gravitational fields, and thermal fields, as well as guiding factors like interface wettability, surface tension-induced self-assembly and template confinement. Researchers have made some progresses on understanding the influence of a single external factor on the behavior of self-assembly, such as ultrasound-assisted self-assembly (Fig. 4a),^34^ light-induced self-assembly (Fig. 4b),^50^ magnetic field-induced self-assembly (Fig. 4c),^48^ template-assisted self-assembly (Fig. 4d)^55,69^ and other efforts.^70^ These insights are highly valuable and provide significant reference value for future work on the precise manipulation and controllable self-assembly of PNCs.

Transforming disordered PNC systems into ordered systems faces multifaceted challenges. These challenges not only involve the intrinsic properties of the materials but also encompass complexities in fabrication processes, theoretical models, and practical applications. In addition to the external stimuli, the exploration of new manipulation strategies is expected to further expand the toolbox for regulating the self-assembly of PNCs. For example, femtosecond laser pulses can induce nonlinear optical effects and transient local heating on ultrafast timescales, thereby triggering rapid ligand rearrangements at nanocrystal surfaces and enabling temporal control over assembly pathways. Plasma fields can activate surfaces, introduce charged species, or locally tune the chemical environment under mild conditions, thereby improving the uniformity and controllability of assembly. Localized electrothermal fields are capable of generating precise temperature gradients at the microscale, driving thermophoretic migration and directional assembly, which is particularly advantageous for constructing anisotropic or heterogeneous architectures. Meanwhile, low- and high-frequency acoustic fields can promote nanocrystal rearrangements through cavitation or mechanical vibration without altering the chemical composition of the system. These external forces are not only experimentally accessible but also capable of perturbing equilibrium states within short or controllable time windows, providing new degrees of freedom for manipulating nanocrystal orientation, ligand dynamics, and packing modes, ultimately enabling dynamic, reversible, and even programmable assembly pathways.

Even more appealing is the concept of synergistic application of multiple external factors. For instance, combining gravitational fields with template-assisted assembly could bias macroscopic directional sedimentation while simultaneously imposing nanoscale periodic constraints. Likewise, coupling optical fields with magnetic or electric fields may facilitate multimodal and programmable patterning of PNC assemblies, thereby achieving unprecedented precision across multiple length scales.

Another frontier lies in the integration of microfluidic platforms into the design of self-assembly systems. Microfluidics inherently provide a confined environment with tunable flow fields, interfacial tensions, and spatiotemporal gradients. When combined with local external fields such as electric or acoustic stimuli, droplet-based synthesis within microfluidic channels can be leveraged to precisely regulate nucleation, growth, and ordered assembly of PNCs. This strategy not only offers scalability and reproducibility but also enables the fabrication of gradient or asymmetric architectures that are challenging to achieve in bulk solution.

### 2.3 Self-assembly of PNCs with morphologies

**Fig. 5:**
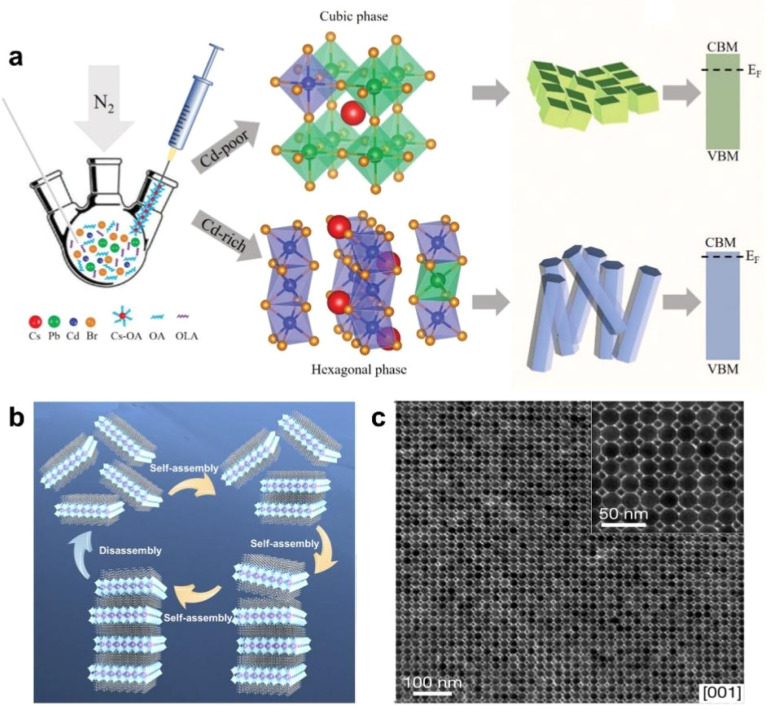
(a) Illustration of the synthetic strategy to modulate the crystallographic structure of alloyed CsPb_1−*x*_Cd_*x*_Br_3_ perovskite NCs by controlling the CdBr_2_/(CdBr_2_ + PbBr_2_) molar ratios in a hot-injection synthetic process.^71^ Copyright 2020, Wiley-VCH. (b) Schematic illustration of the layer-by-layer, self-assembly of C_8_H_17_NH_3_-capped CsPb_2_Br_7_ nanosheets into layered (C_8_H_17_NH_3_)_2_CsPb_2_Br_7_ superlattice nanocrystals.^72^ Copyright 2019, American Chemical Society. (c) TEM images of a binary ABO_3_-type superlattice assembled CsPbBr_3_ and Fe_3_O_4_ nanocrystals.^56^ Copyright 2021, Springer Nature.

As a highly malleable ionic crystal, the ductility on multiple crystal planes gives its strong morphological plasticity. According to existing reports, the morphology of nanocrystals has been shaped into variously sized cubes,^73–75^ nanorods,^76,77^ nanosheets,^78^ nanowires,^79^ and even polyhedra with multiple planes (Fig. 5a).^71^ However, the self-assembly processes and mechanisms of these nanostructures with diverse morphologies and sizes have rarely been reported. Research on the self-assembly of nanocrystals with different morphologies remains at a very early stage, requiring extensive further investigation by researchers. Furthermore, when PNCs are incorporated as a component, they can form novel systems by combining with other diverse material systems (Fig. 5b and c).^56,72^ These new superlattices exhibit distinct packing arrangements and morphological characteristics, consequently yielding unique material properties. It is foreseeable that the packing modes, formation mechanisms, and the exceptional optoelectronic properties of such multicomponent superlattices will emerge as critically important research directions in the future.

## 3. Characterization of OPNC structures

The structural ordering within PNC assemblies significantly influences their optoelectronic properties, thereby demanding advanced characterization techniques to uncover the correlations between structural order and functional properties. This chapter presents a comprehensive review of multidimensional characterization methods for OPNCs, encompassing real-space imaging, reciprocal-space imaging, and spectroscopic measurements. Together, these approaches yield complementary insights into the structure–property relationships, facilitating a deeper understanding of the underlying mechanisms.

### 3.1 Real-space imaging

**Fig. 6:**
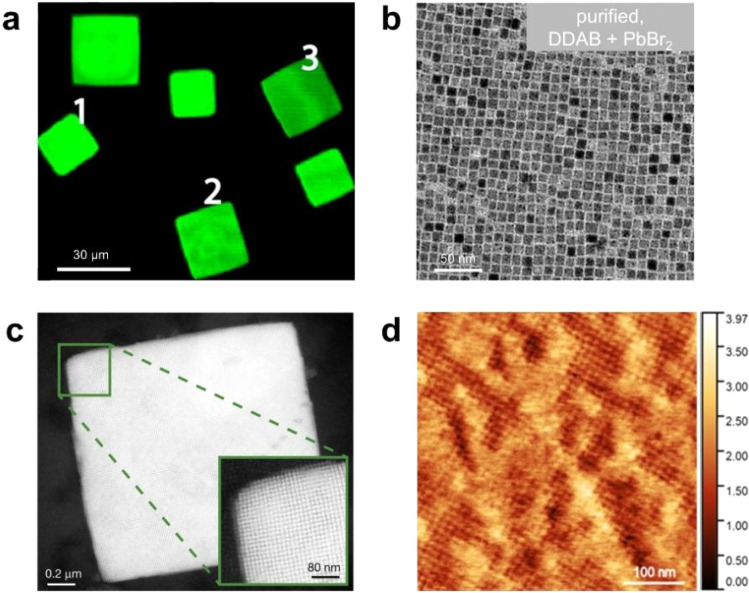
(a) Confocal microscopy image of CsPbBr_3_ superlattices.^80^ Copyright 2018, American Chemical Society. (b) TEM imaging of monodisperse CsPbBr_3_ nanocrystals treated with DDAB ligand.^81^ Copyright 2019, American Chemical Society. (c) HAADF-STEM image of a single superlattice of CsPbBr_3_ PNCs. Inset: enlarged view of the marked area, exhibiting the individual PNCs.^41^ Copyright 2018, Springer Nature. (d) AFM image of a monolayer film a PNC surface with an RMS of 4 Å.^82^ Copyright 2018, American Chemical Society.

Due to their typical width of several micrometers, OPNC assemblies can be directly visualized using confocal microscopy when stimulated with UV or blue light. For example, under 488 nm excitation, confocal imaging captures green photoluminescence from square-shaped CsPbBr_3_ superlattices (Fig. 6a).^80^ The formation of such assemblies can be directly characterized using transmission electron microscopy (TEM) and high-angle annular dark-field scanning transmission electron microscopy (HAADF-STEM).^40^ For instance, PNCs passivated with didodecylammonium bromide (DDAB) exhibit high size uniformity, enabling the organization of individual nanocrystals into well-defined architectures (Fig. 6b).^81^ Through toluene solvent evaporation, three-dimensional superlattices consisting of highly ordered and well-separated nanocrystals have been successfully fabricated (Fig. 6c).^41^ Additionally, atomic force microscopy (AFM) plays a critical role in probing surface morphology, uniformity, and nanoscale features of self-assembled PNC films.^82,83^ The use of short-chain thiocyanate (SCN^−^) ligands, for example, promotes the formation of uniformly sized nanocrystals, resulting in highly ordered thin films with exceptionally smooth surfaces, exhibiting a root-mean-square roughness as low as 4 Å (Fig. 6d).^82^

### 3.2 Reciprocal space imaging

**Fig. 7:**
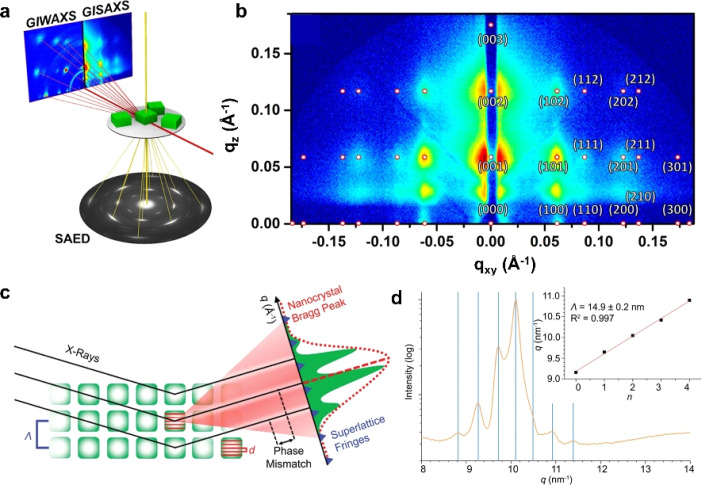
(a) Working principles of electron microscopy, GISAXS, and GIWAXS techniques. (b) GISAXS pattern of CsPbBr_3_ superlattices indexed according to a simple-cubic symmetry. (c) X-ray scattering method for illustrating nanocrystal Bragg peaks and superlattice fringes.^84^ Copyright 2021, American Chemical Society. (d) XRD peak splitting within a small-angle region in FASnI_3_ superlattices.^85^ Copyright 2021, Springer Nature.

In a typical measurement, a high-energy electron beam is used to penetrate the sample. Through electromagnetic interactions with atomic nuclei and electrons, the incident electrons are scattered at various angles, forming two- or three-dimensional images. Since the scattering angle correlates with the density and thickness of the sample, high-resolution structural information can be obtained. Complementing real-space imaging *via* TEM, reciprocal-space analysis techniques such as Selected Area Electron Diffraction (SAED) and Fast Fourier Transform allow precise identification of periodic structures at the nanoscale and atomic levels (Fig. 7a).^84^ Beyond electron-based techniques, synchrotron-based razing-incidence small-angle X-ray scattering (GISAXS) and grazing-incidence wide-angle X-ray scattering (GIWAXS) have become powerful tools for characterizing structurally OPNC assemblies (Fig. 7a).^84^ GISAXS is sensitive to small-angle scattering, making it suitable for probing larger-scale periodicities, whereas GIWAXS covers wider angles and yields more diffraction spots, thereby revealing atomic and molecular-scale ordering. Thus, due to the periodic arrangement in OPNC assemblies, a series of the aforementioned patterns can be obtained within certain detection angle ranges. For example, a GISAXS scattering image of a superlattice made of CsPbBr_3_ nanocrystals shows nearly perfect periodic patterns, indicating their high crystalline quality (Fig. 7b).^84^ When X-rays interact with superlattices, they are diffracted by the periodic atomic planes of the constituent nanocrystals, which function as nanoscale diffraction gratings. Isolated nanocrystals yield a diffraction pattern characterized by intrinsic size broadening, typically appearing as a diffuse peak with low-intensity subsidiary fringes (Fig. 7c).^84^ In contrast, long-range periodic organization of nanocrystals into a superlattice generates a set of sharp diffraction features at defined angular positions. Due to the considerable experimental demands of techniques such as GISAXS and GIWAXS, high-resolution laboratory wide-angle X-ray diffraction presents a more practical approach for assessing superlattice order. A critical signature of a well-ordered superlattice is the observation of peak splitting in the low-angle region (Fig. 7d).^85^

### 3.3 Spectroscopic proxies

**Fig. 8:**
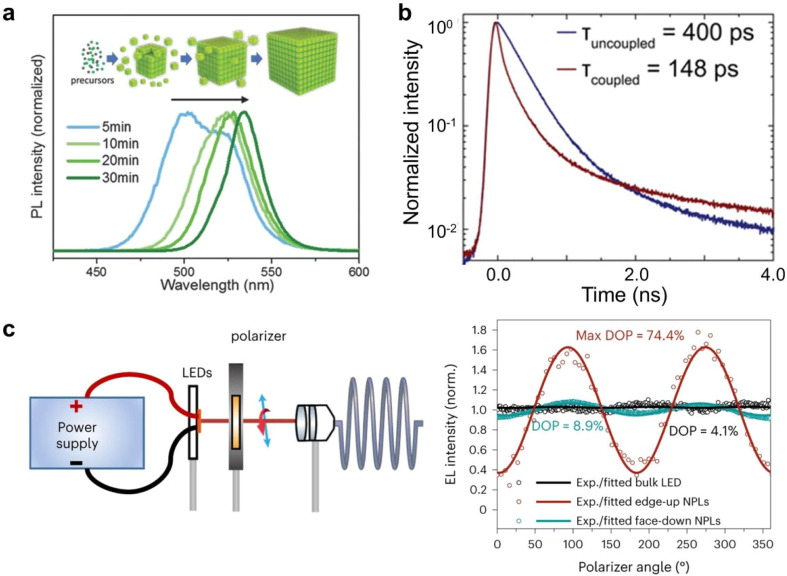
(a) Reversible spectral redshift in superlattice formation from dispersed nanocrystals.^34^ Copyright 2018, Wiley-VCH. (b) Time-resolved photoluminescence decay of quantum dots and coupled quantum dot superlattices.^41^ Copyright 2018, Springer Nature. (c) Set-up used to measure the polarized EL and polarization dependence of the electroluminescence for CsPbI_3_ nanoplatelet superlattices and bulk FAPbI_3_ LEDs.^86^ Copyright 2024, Springer Nature.

The strong electronic coupling between closely packed PNCs in ordered assemblies leads to the formation of non-localized, extended electronic bands. This collective electronic behavior results in a reduced effective band gap, manifested as a distinct red-shift in the photoluminescence emission spectra. For instance, extended aging during self-assembly promotes the formation of larger superlattices, wherein enhanced interparticle interactions amplify this redshift effect due to stronger electronic coupling among the constituent nanocrystals (Fig. 8a).^34^ A reduction in exciton lifetime is observed when PNCs form closely packed films, superlattices, or dimeric structures (Fig. 8b).^41^ Furthermore, the self-assembly process often imparts anisotropic structural features to PNC superlattices, which in turn induce direction-dependent optoelectronic properties. Under electric field excitation, aligned perovskite nanoplatelets exhibit strongly polarized electroluminescence. The emission intensity reaches maximum when the detection polarization is oriented in-plane with the nanoplatelets, whereas out-of-plane polarization yields significantly attenuated emission (Fig. 8c).^86^ This pronounced luminescence anisotropy can be quantitatively assessed using polarization-resolved electroluminescence spectroscopy, providing critical insights into the orientation of transition dipole moments and the degree of nanocrystal alignment within the superlattice.

## 4. Prospective potential applications of OPNCs

At present, research on ordered nanocrystalline material systems has shown many potential applications, such as high-performance photodetection, LEDs, low-threshold lasers. Due to the coherent interactions among nanocrystals, collective emission phenomena such as superfluorescence and Burnham–Chiao ringing can be expected.^38,87^ The emergence of distinctive optical phenomena arising from the collective behavior of a large number of nanocrystals which cannot be achieved by simply defect reduction of a disordered system. The generation of this coherence requires the formation of superlattices through dense and ordered packing, as well as an inert and cryogenic environment to suppress thermal dephasing.^88^ Interestingly, Melike *et al.* reported at higher temperatures exciton–lattice interactions lead to a new electronically and structurally entangled coherent extended solitonic state beyond a critical polaron density. While macroscopic quantum coherence among excitons simultaneously emerges.^88^ Recombination of excitons in this state culminates in superfluorescence at high temperatures which is different superfluorescence with OPNC systems, this gives a new insight of superfluorescence. However, it is notable that current research on superlattices primarily focuses on solution-mediated, randomly formed superlattices. Due to constraints in fabrication techniques and material development, the preparation, characterization, and study of size, position and morphology-controlled superlattices remain scarce. This represents both the core challenge and the key issues for controllable self-assembly technology.

### 4.1 Pixelated and patterned lighting applications

**Fig. 9:**
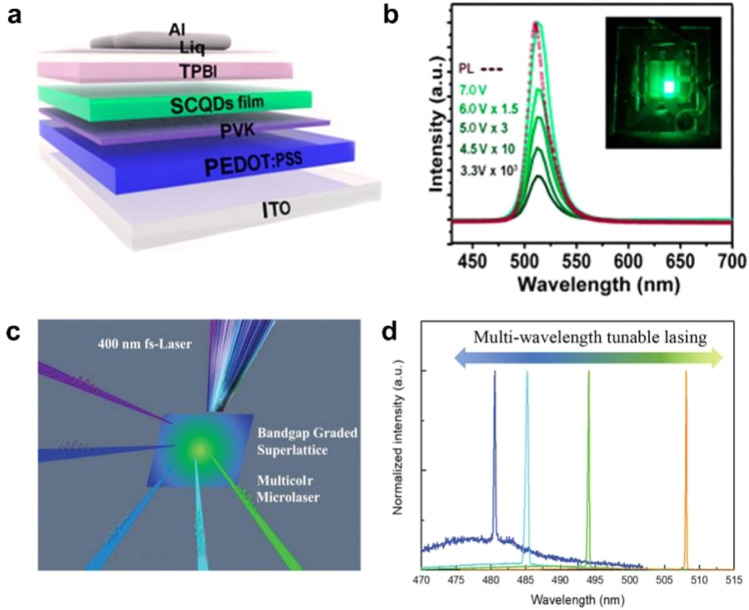
(a) Schematic of CsPbBr_3_ SCQDs based PeLEDs. (b) EL spectra measured with various voltages (inset: photograph of the corresponding device operated under 4 V).^90^ Copyright 2018, American Chemical Society. (c) Schematic of optically pumping lasing experiments with 400 nm fs-laser (≈40 fs, 10 kHz) as the excitation source. (d) Multi-wavelength tunable lasing of typical sites on alloy superlattice.^91^ Copyright 2022, Wiley-VCH.

The application of pixelated and patterned PNCs in LEDs has become relatively mature.^13,89^ Traditional PNCs are coated with long-chain ligands on their surface, which significantly impedes charge carrier transport. Additionally, their environmental sensitivity substantially reduces device lifetime. The introduction of perovskite superlattices can alleviate these issues to some extent. Although researchers have already used OPNCs to achieve some applications on LEDs (Fig. 9a and b),^90^ due to limitations in fabrication techniques, researchers still lack effective methods to control the assembly position, orientation, and three-dimensional morphology of these superlattices.

A promising strategy involves treating superlattices with controlled morphology, position, and orientation as independent pixels, which holds potential for both photoluminescent and electroluminescent devices. This approach not only enhances charge carrier transport capabilities compared to disordered PNC emitting layers but also significantly improves stability through the protective framework of the superlattice structure. Furthermore, pixelated superlattice arrays enable higher degrees of freedom and controllable patterning applications, demonstrating crucial potential in anti-counterfeiting, recognition, data storage, and computing technologies.

### 4.2 Advanced low-threshold lasers

PNCs demonstrated significant advantages in low-threshold lasers and resonator cavity applications,^92–94^ and the highly ordered assemblies fundamentally address the key limitations of disordered PNC systems. Fig. 9c and d shows the typical lasing spectra of a single CsPbBr_3−3*x*_Cl_3*x*_ alloy superlattice recorded at a high pump density. Tunable lasing at 480, 485, 494, and 508 nm can be clearly observed.^91^ Compared to randomly stacked nanocrystals, superlattices form miniband structures through strong quantum coupling, which greatly enhances carrier transport and energy transfer while significantly reducing non-radiative recombination losses. Simultaneously, their periodic arrangement spontaneously generates a distributed feedback (DFB) effect, functioning as a built-in photonic crystal resonator that effectively improves optical feedback and mode selection capabilities. These combined characteristics enable lower lasing thresholds and higher slope efficiency. Furthermore, the superlattice structure provides physical protection to the internal nanocrystals, markedly enhancing environmental stability. With the advancement of controllable fabrication techniques for perovskite superlattices, the future will see the development of high-performance devices such as laser arrays and photonic chips, as well as more complex laser architectures based on PNC superlattices.

### 4.3 Advanced solar cell and photodetection

**Fig. 10:**
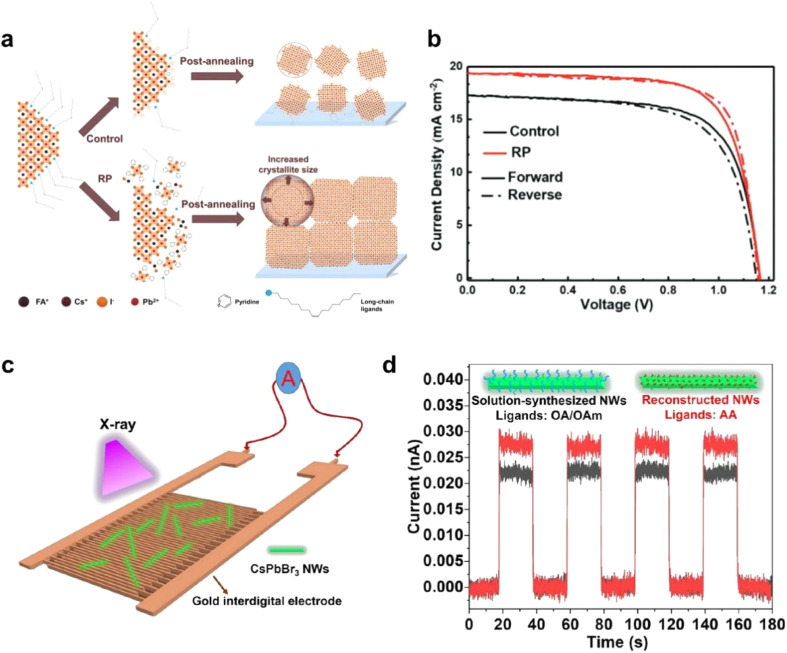
(a) The schematic of the control method and RP process method. (b) *J*–*V* curves of control and RP device under AM 1.5G, 100 mW cm^−2^ simulated solar illumination.^95^ Copyright 2023, Wiley-VCH. (c) Schematic of the fabricated photodetector based on CsPbBr_3_ NWs. (d) Photocurrent response of devices based on CsPbBr_3_ NWs from solution-based synthesis (capped with OA/OAm) and reconstructed synthesis (capped with AA) under X-ray illumination (a bias voltage of 1 V).^96^ Copyright 2022, American Chemical Society.

Perovskite solar cells^94,99–103^ and photodetectors^104–106^ have demonstrated remarkable performance and potential in practical applications, particularly in the case of NC-based devices.^97,98^ Although the efficiency of NC perovskite solar cells has been continuously improving, their stability and interfacial charge transport remain significant challenges. These issues impose higher demands on the stability of the nanocrystal functional layers and the efficiency of carrier transport. One of the most promising strategies to address these problems is the ordered assembly of PNCs, which facilitates more effective contact between PNCs themselves and between PNCs and functional layers. This approach can substantially enhance carrier transport among PNCs and improve interfacial charge transfer across functional layers. Optimizing the properties of the interfaces in contact with nanocrystals is an effective method to improve both NC alignment and charge transport performance. As Fig. 10a and b shows, Yang *et al.* proposed a re-assembling (RP) strategy for PNC films to overcome charge confinement. This is achieved by enlarging crystallites and stripping long-chain ligands. Notably, the RP process induces a preferential orientation (90° azimuth) in the films, in contrast to the random orientation of the controls. These structural modifications collectively facilitate exciton dissociation and enhance carrier extraction, thus boosting the overall performance of the solar cells.^95^

PNCs have also gained significant attention for photodetection applications, where OPNC assemblies offer distinct advantages. The highly periodic structure promotes efficient carrier transport and collection, which is crucial for achieving high-speed photoresponse (Fig. 10c). Furthermore, the enhanced inter-dot coupling within assembled structures effectively reduces trap-assisted recombination, leading to lower noise currents and improved detection sensitivity (Fig. 10d). These integrated benefits position well-structured PNC assemblies as promising candidates for next-generation photodetection technologies.

## 5. Outlook

**Fig. 11:**
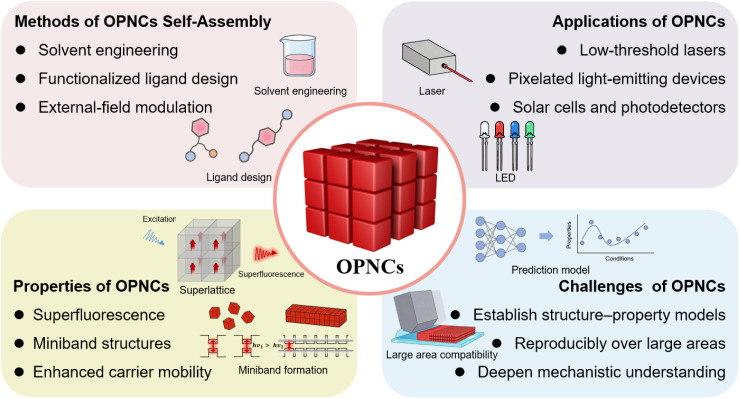
Schematic diagram of OPNC self-assembly methods, properties, applications and challenges.

Overall, this review systematically elaborates on the self-assembly methods and mechanisms, unique properties, optoelectronic applications, and future challenges of OPNCs. As summarized in Fig. 11, the key points have been outlined to provide a clear overview. Notably, research on controllable self-assembly of PNCs is still in its infancy, and several key issues must be addressed before OPNCs can be fully exploited. A first priority is to deepen the mechanistic understanding of self-assembly driving forces. While the roles of Brownian motion, van der Waals attraction, ligand interactions, and external fields have been identified, quantitative correlations between these factors and long-range order remain unclear.

Second, progress of OPNC as a new optoelectronic platform will require the integration of multiple strategies already explored in isolation. For example, solvent polarity control, functionalized ligand design, and external-field modulation have each shown promise, but their combined or synergistic application could yield higher precision and reproducibility in ordering. Similarly, extending self-assembly beyond cubic nanocrystals to multiple morphologies and multicomponent systems may unlock new packing modes and collective properties.

Third, translating OPNC structures into devices demands greater emphasis on stability and scalability. Ordered assemblies must maintain structural integrity and optoelectronic performance under illumination, electrical bias, and environmental exposure. At the same time, solution-mediated and substrate-mediated assembly methods need to evolve toward scalable and controllable processing that can generate OPNCs reproducibly over large areas.^107^

Future progress will also depend critically on establishing quantitative structure–property correlation frameworks capable of predicting how specific geometric and chemical parameters translate into functional performance. Although existing studies have revealed the influence of interparticle distance, orientational order, ligand identity, and lattice coherence on electronic coupling and exciton transport, a unified and quantitative model connecting these structural descriptors to measurable optoelectronic outputs—such as miniband formation, carrier mobility, exciton diffusion length, and lasing threshold—remains largely undeveloped. Integrating multiscale theoretical modeling with advanced scattering, ultrafast spectroscopy, and data-driven analytical methods could enable predictive design rules for OPNC superlattices. Establishing such frameworks will be essential for transitioning from empirical assembly strategies toward rational, programmable, and ultimately device-oriented OPNC engineering.

Addressing these challenges will not only clarify the structure–property relationships in OPNC assemblies but also accelerate their application in photovoltaic, light-emitting devices, lasers, and photodetectors. In this way, controllable self-assembly can transform OPNCs from disordered colloids into ordered materials with practical and reliable optoelectronic functionality.

## Author contributions

L. Z. and H. L. contribute equally to this work.

## Conflicts of interest

There are no conflicts to declare.

## Data Availability

No primary research results, software or code have been included and no new date were generated or analysed as part of this review.

## References

[cit1] Zhang X., Huang H., Zhao C., Jin L., Lee C., Li Y., Ko D.-H., Ma W., Wu T., Yuan J. (2024). Nat. Energy.

[cit2] Jang K. Y., Hwang S. Y., Woo S.-J., Yoon E., Park C.-Y., Kim S. Y., Kim D.-H., Kim H., Park J., Sargent E. H., Lee T.-W. (2024). Adv. Mater..

[cit3] Kong L., Sun Y., Zhao B., Ji K., Feng J., Dong J., Wang Y., Liu Z., Maqbool S., Li Y., Yang Y., Dai L., Lee W., Cho C., Stranks S. D., Friend R. H., Wang N., Greenham N. C., Yang X. (2024). Nature.

[cit4] Zhang F., Yang Y., Gao Y., Wang D., Dong W., Lu P., Wang X., Lu M., Wu Y., Chen P., Hu J., Yang X., Liu D., Xu L., Dong B., Wu Z., Zhang Y., Song H., Bai X. (2024). Nano Lett..

[cit5] Yoo J., Lee K., Yang U. J., Song H. H., Jang J. H., Lee G. H., Bootharaju M. S., Kim J. H., Kim K., Park S. I., Seo J. D., Li S., Yu W. S., Kwon J. I., Song M. H., Hyeon T., Yang J., Choi M. K. (2024). Nat. Photonics.

[cit6] Moon J., Mehta Y., Gundogdu K., So F., Gu Q. (2024). Adv. Mater..

[cit7] Jana A., Cho S., Patil S. A., Meena A., Jo Y., Sree V. G., Park Y., Kim H., Im H., Taylor R. A. (2022). Mater. Today.

[cit8] Kim J. S., Heo J.-M., Park G.-S., Woo S.-J., Cho C., Yun H. J., Kim D.-H., Park J., Lee S.-C., Park S.-H., Yoon E., Greenham N. C., Lee T.-W. (2022). Nature.

[cit9] Yang Z., Yao J., Xu L., Fan W., Song J. (2024). Nat. Commun..

[cit10] Luo Z., Yin W., Wang J., Hua Y., Zhou Z., Zhang W., Chen S., Zhang X., Zheng W. (2025). Adv. Funct. Mater..

[cit11] Singh S., Anandan P. R., Shahrokhi S., Nguyen H., Lin C.-H., Hu L., Guan X., Younis A., Sharma P., Seidel J., Wu T. (2025). ACS Appl. Mater. Interfaces.

[cit12] Cheng H., Zheng Y., Lou Y., Sun M., Zhang G., Wang H., Wu T., Bai Y., Shao Y. (2025). Adv. Electron. Mater..

[cit13] Yamada Y., Tsung C.-K., Huang W., Huo Z., Habas S. E., Soejima T., Aliaga C. E., Somorjai G. A., Yang P. (2011). Nat. Chem..

[cit14] Xie M., Guo J., Bi C., Zhang Y., Li H., Zhang L., Zhang L., Zhang X., Zheng W., Tian J. (2024). ACS Energy Lett..

[cit15] Xie M., Guo J., Zhang X., Bi C., Zhang L., Chu Z., Zheng W., You J., Tian J. (2022). Nano Lett..

[cit16] Dong B., Jiang Y., Guan X., Zheng X., Yin S., Gong B., Wan T., Mei T., Chen F., Li Z., Li M., Yang A., Ahmad O., Chae W., Han J., Chen C., Gao L., Kim J., Lin C.-H., Wang G., Lu Y., Huang S., Wu T., Chu D., Hu L. (2025). ACS Energy Lett..

[cit17] Hu L., Duan L., Yao Y., Chen W., Zhou Z., Cazorla C., Lin C.-H., Guan X., Geng X., Wang F., Wan T., Wu S., Cheong S., Tilley R. D., Liu S., Yuan J., Chu D., Wu T., Huang S. (2022). Adv. Sci..

[cit18] Duan L., Hu L., Guan X., Lin C.-H., Chu D., Huang S., Liu X., Yuan J., Wu T. (2021). Adv. Energy Mater..

[cit19] Wu X., Jing Y., Zhong H. (2024). Adv. Mater..

[cit20] Sun J.-K., Huang S., Liu X.-Z., Xu Q., Zhang Q.-H., Jiang W.-J., Xue D.-J., Xu J.-C., Ma J.-Y., Ding J., Ge Q.-Q., Gu L., Fang X.-H., Zhong H.-Z., Hu J.-S., Wan L.-J. (2018). J. Am. Chem. Soc..

[cit21] Sun S., Huang P., Wu X., Chen C., Hu X., Bai Z., Pushkarev A., Zhong H. (2024). J. Phys. Chem. C.

[cit22] Shi Y., Yuan L., Liu Z., Lu Y., Yuan B., Shen W., Xue B., Zhang Y., Qian Y., Li F., Zhang X., Liu Y., Wang Y., Wang L., Yuan J., Liao L.-S., Yang B., Yu Y., Ma W. (2022). ACS Nano.

[cit23] Cao J., Zhang X., Miao Y., Li W., Zeng X., Yang S., Yan C., Lu J., Yang W. (2024). Matter.

[cit24] Li Y., Wang D., Yang Y., Ding C., Hu Y., Liu F., Wei Y., Liu D., Li H., Shi G., Chen S., Li H., Fuchimoto A., Tosa K., Hiroki U., Hayase S., Wei H., Shen Q. (2024). J. Am. Chem. Soc..

[cit25] Zhang Y., Wen D., Wang M., Zhang T., Cao K., Chen R. (2025). J. Inf. Disp..

[cit26] Pan Q., Zhao Q., Wei P., Li G. (2025). ChemSusChem.

[cit27] Wang Y.-K., Wan H., Teale S., Grater L., Zhao F., Zhang Z., Duan H.-W., Imran M., Wang S.-D., Hoogland S., Liao L.-S. (2024). Nature.

[cit28] Nawrocki J., Anilkumar V., Jia G., Mahapatra A., Bernatowicz P., Dellith J., Gayatri, Raczyński M., Karmakar A., Yadav P., Akin S., Dietzek-Ivanšić B., Molas M. R., Plentz J., Prochowicz D. (2025). J. Mater. Chem. A.

[cit29] He S., Lin W., Yu D., Shi J., Yin Z., Sun C., Liu H., Zhang C., Yuan J., Bai S., Xiao S., Long G., Yuan M., Jiang Y., Chen Y., Song Q. (2025). Nat. Commun..

[cit30] Wang Z., Fu R., Li F., Xie H., He P., Sha Q., Tang Z., Wang N., Zhong H. (2021). Adv. Funct. Mater..

[cit31] Wu X., Ji H., Yan X., Zhong H. (2022). Nat. Nanotechnol..

[cit32] Shi L., Meng L., Jiang F., Ge Y., Li F., Wu X., Zhong H. (2019). Adv. Funct. Mater..

[cit33] Zhou Q., Bai Z., Lu W.-G., Wang Y., Zou B., Zhong H. (2016). Adv. Mater..

[cit34] Tong Y., Yao E.-P., Manzi A., Bladt E., Wang K., Döblinger M., Bals S., Müller-Buschbaum P., Urban A. S., Polavarapu L., Feldmann J. (2018). Adv. Mater..

[cit35] Liao S., Yang Z., Lin J., Wang S., Zhu J., Chen S., Huang F., Zheng Y., Chen D. (2023). Adv. Funct. Mater..

[cit36] Guo Y., Li X., Chen B., Tang Y., Wang J., Lu H., Guo C., Tong S.-Y. (2024). Mater. Today Energy.

[cit37] Liu Z., Qin X., Chen Q., Jiang T., Chen Q., Liu X. (2023). Adv. Mater..

[cit38] Li Y., Zhang F. (2024). J. Energy Chem..

[cit39] Baranov D., Fieramosca A., Yang R. X., Polimeno L., Lerario G., Toso S., Giansante C., Giorgi M. D., Tan L. Z., Sanvitto D., Manna L. (2021). ACS Nano.

[cit40] Liu J., Zheng X., Mohammed O. F., Bakr O. M. (2022). Acc. Chem. Res..

[cit41] Rainò G., Becker M. A., Bodnarchuk M. I., Mahrt R. F., Kovalenko M. V., Stöferle T. (2018). Nature.

[cit42] Scheibner M., Schmidt T., Worschech L., Forchel A., Bacher G., Passow T., Hommel D. (2007). Nat. Phys..

[cit43] Douglas S. M., Dietz H., Liedl T., Högberg B., Graf F., Shih W. M. (2009). Nature.

[cit44] Boles M. A., Engel M., Talapin D. V. (2016). Chem. Rev..

[cit45] Bishop K. J. M., Wilmer C. E., Soh S., Grzybowski B. A. (2009). Small.

[cit46] Steiner T. (2002). Angew. Chem., Int. Ed..

[cit47] Noh S. H., Jeong W., Lee K. H., Yang H. S., Suh E. H., Jung J., Park S. C., Lee D., Jung I. H., Jeong Y. J., Jang J. (2023). Adv. Funct. Mater..

[cit48] Singh G., Chan H., Baskin A., Gelman E., Repnin N., Král P., Klajn R. (2014). Science.

[cit49] Ravi V. K., Scheidt R. A., DuBose J., Kamat P. V. (2018). J. Am. Chem. Soc..

[cit50] Liu J., Song K., Shin Y., Liu X., Chen J., Yao K. X., Pan J., Yang C., Yin J., Xu L.-J., Yang H., El-Zohry A. M., Xin B., Mitra S., Hedhili M. N., Roqan I. S., Mohammed O. F., Han Y., Bakr O. M. (2019). Chem. Mater..

[cit51] Zhong C., Yu K., Qie Y., Yu Y., Lu Y., Deng G., Guo T., Hu H., Li F. (2025). Adv. Funct. Mater..

[cit52] Li H., Zhang J., Wen W., Zhao Y., Gao H., Ji B., Wang Y., Jiang L., Wu Y. (2025). Nat. Commun..

[cit53] Dang Z., Dhanabalan B., Castelli A., Dhall R., Bustillo K. C., Marchelli D., Spirito D., Petralanda U., Shamsi J., Manna L., Krahne R., Arciniegas M. P. (2020). Nano Lett..

[cit54] Chaouiki A., Chafiq M., Ko Y. G. (2024). Mater. Sci. Eng., R.

[cit55] Jia Y., Li H., Guo N., Li F., Li T., Ma H., Zhao Y., Gao H., Wang D., Feng J., He Z., Jiang L., Wu Y. (2025). Nat. Commun..

[cit56] Cherniukh I., Rainò G., Stöferle T., Burian M., Travesset A., Naumenko D., Amenitsch H., Erni R., Mahrt R. F., Bodnarchuk M. I., Kovalenko M. V. (2021). Nature.

[cit57] Ji C., Zeng F., Xu W., Zhu M., Yu H., Yang H., Peng Z. (2025). Adv. Mater..

[cit58] Soetan N., Erwin W. R., Tonigan A. M., Walker D. G., Bardhan R. (2017). J. Phys. Chem. C.

[cit59] Chen L., Hu Y., Zhou B., Dong H., Mou N., He J., Yang Z., Li J., Zhu Z., Zhang L. (2022). Adv. Mater. Interfaces.

[cit60] Sun J.-K., Huang S., Liu X.-Z., Xu Q., Zhang Q.-H., Jiang W.-J., Xue D.-J., Xu J.-C., Ma J.-Y., Ding J., Ge Q.-Q., Gu L., Fang X.-H., Zhong H.-Z., Hu J.-S., Wan L.-J. (2018). J. Am. Chem. Soc..

[cit61] Mehetor S. K., Ghosh H., Pradhan N. (2019). ACS Energy Lett..

[cit62] Huang Y., Xiao L., Wang L., Zhu G., Yin S., Cheng S., Gu H., Du Q., Yeow E. K. L., Wong T. N., Sun H. (2020). Appl. Mater. Today.

[cit63] Cherniukh I., Sekh T. V., Rainò G., Ashton O. J., Burian M., Travesset A., Athanasiou M., Manoli A., John R. A., Svyrydenko M., Morad V., Shynkarenko Y., Montanarella F., Naumenko D., Amenitsch H., Itskos G., Mahrt R. F., Stöferle T., Erni R., Kovalenko M. V., Bodnarchuk M. I. (2022). ACS Nano.

[cit64] Bertoncello P., Wilson N. R., Unwin P. R. (2007). Soft Matter.

[cit65] Yamauchi M., Masuo S. (2020). Chem.–Eur. J..

[cit66] Kim Y., Song R., Hao J., Zhai Y., Yan L., Moot T., Palmstrom A. F., Brunecky R., You W., Berry J. J., Blackburn J. L., Beard M. C., Blum V., Luther J. M. (2022). Adv. Funct. Mater..

[cit67] Ruan L., Shen W., Wang A., Zhou Q., Zhang H., Deng Z. (2017). Nanoscale.

[cit68] Tang B., Wang S., Liu H., Mou N., Portniagin A. S., Chen P., Wu Y., Gao X., Lei D., Rogach A. L. (2024). Adv. Opt. Mater..

[cit69] Feng J., Qiu Y., Jiang L., Wu Y. (2022). Adv. Mater..

[cit70] Chandran B R R., Raj A., Jayakrishnan R. (2023). J. Sci.: Adv. Mater. Devices.

[cit71] Guo J., Fu Y., Lu M., Zhang X., Kershaw S. V., Zhang J., Luo S., Li Y., Yu W. W., Rogach A. L., Zhang L., Bai X. (2020). Adv. Sci..

[cit72] Liu Y., Siron M., Lu D., Yang J., Dos Reis R., Cui F., Gao M., Lai M., Lin J., Kong Q., Lei T., Kang J., Jin J., Ciston J., Yang P. (2019). J. Am. Chem. Soc..

[cit73] Hudait B., Dutta S. K., Pradhan N. (2020). ACS Energy Lett..

[cit74] Wang K.-H., Yang J.-N., Ni Q.-K., Yao H.-B., Yu S.-H. (2018). Langmuir.

[cit75] Yang D., Li X., Zhou W., Zhang S., Meng C., Wu Y., Wang Y., Zeng H. (2019). Adv. Mater..

[cit76] Yin T., Fang Y., Chong W. K., Ming K. T., Jiang S., Li X., Kuo J.-L., Fang J., Sum T. C., White T. J., Yan J., Shen Z. X. (2018). Adv. Mater..

[cit77] Xiao X., Li Y., Xie R.-J. (2020). Nanoscale.

[cit78] Ji C., Wang S., Wang Y., Chen H., Li L., Sun Z., Sui Y., Wang S., Luo J. (2020). Adv. Funct. Mater..

[cit79] Pan A., Jurow M. J., Wu Y., Jia M., Zheng F., Zhang Y., He L., Liu Y. (2019). ACS Appl. Nano Mater..

[cit80] Imran M., Ijaz P., Baranov D., Goldoni L., Petralanda U., Akkerman Q., Abdelhady A. L., Prato M., Bianchini P., Infante I., Manna L. (2018). Nano Lett..

[cit81] Bodnarchuk M. I., Boehme S. C., Ten Brinck S., Bernasconi C., Shynkarenko Y., Krieg F., Widmer R., Aeschlimann B., Günther D., Kovalenko M. V., Infante I. (2019). ACS Energy Lett..

[cit82] Patra B. K., Agrawal H., Zheng J.-Y., Zha X., Travesset A., Garnett E. C. (2020). ACS Appl. Mater. Interfaces.

[cit83] Jin X., Zhang X., Fang H., Deng W., Xu X., Jie J., Zhang X. (2018). Adv. Funct. Mater..

[cit84] Toso S., Baranov D., Altamura D., Scattarella F., Dahl J., Wang X., Marras S., Alivisatos A. P., Singer A., Giannini C., Manna L. (2021). ACS Nano.

[cit85] Dai L., Deng Z., Auras F., Goodwin H., Zhang Z., Walmsley J. C., Bristowe P. D., Deschler F., Greenham N. C. (2021). Nat. Photonics.

[cit86] Ye J., Ren A., Dai L., Baikie T. K., Guo R., Pal D., Gorgon S., Heger J. E., Huang J., Sun Y., Arul R., Grimaldi G., Zhang K., Shamsi J., Huang Y.-T., Wang H., Wu J., Koenderink A. F., Torrente Murciano L., Schwartzkopf M., Roth S. V., Müller-Buschbaum P., Baumberg J. J., Stranks S. D., Greenham N. C., Polavarapu L., Zhang W., Rao A., Hoye R. L. Z. (2024). Nat. Photonics.

[cit87] Heinzen D. J., Thomas J. E., Feld M. S. (1985). Phys. Rev. Lett..

[cit88] Biliroglu M., Türe M., Ghita A., Kotyrov M., Qin X., Seyitliyev D., Phonthiptokun N., Abdelsamei M., Chai J., Su R., Herath U., Swan A. K., Temnov V. V., Blum V., So F., Gundogdu K. (2025). Nature.

[cit89] Zhang Q., Zhang D., Liao Z., Cao Y. B., Kumar M., Poddar S., Han J., Hu Y., Lv H., Mo X., Srivastava A. K., Fan Z. (2025). Adv. Mater..

[cit90] Yuan S., Wang Z.-K., Zhuo M.-P., Tian Q.-S., Jin Y., Liao L.-S. (2018). ACS Nano.

[cit91] Chen L., Zhou B., Hu Y., Dong H., Zhang G., Shi Y., Zhang L. (2022). Adv. Opt. Mater..

[cit92] Yan D., Shi T., Zang Z., Zhou T., Liu Z., Zhang Z., Du J., Leng Y., Tang X. (2019). Small.

[cit93] Chen J., Du W., Shi J., Li M., Wang Y., Zhang Q., Liu X. (2020). InfoMat.

[cit94] Zou C., Ren Z., Hui K., Wang Z., Fan Y., Yang Y., Yuan B., Zhao B., Di D. (2025). Nature.

[cit95] Yang W., Jo S.-H., Tang Y., Park J., Ji S. G., Cho S. H., Hong Y., Kim D.-H., Park J., Yoon E., Zhou H., Woo S.-J., Kim H., Yun H. J., Lee Y. S., Kim J. Y., Hu B., Lee T.-W. (2023). Adv. Mater..

[cit96] Zhao C., He Z., Wangyang P., Tan J., Shi C., Pan A., He L., Liu Y. (2022). ACS Appl. Nano Mater..

[cit97] Zhao C., Li D., Zhang X., Huang H., Cazorla C., Zhao X., Li H., Chen Y., Zhu W., Wu T., Yuan J. (2025). Adv. Mater..

[cit98] Zhang X., Huang H., Zhao C., Yuan J. (2025). Chem. Soc. Rev..

[cit99] Jia L., Xia S., Li J., Qin Y., Pei B., Ding L., Yin J., Du T., Fang Z., Yin Y., Liu J., Yang Y., Zhang F., Wu X., Li Q., Zhao S., Zhang H., Li Q., Jia Q., Liu C., Gu X., Liu B., Dong X., Liu J., Liu T., Gao Y., Yang M., Yin S., Ru X., Chen H., Yang B., Zheng Z., Zhou W., Dou M., Wang S., Gao S., Chen L., Qu M., Lu J., Fang L., Wang Y., Deng H., Yu J., Zhang X., Li M., Lang X., Xiao C., Hu Q., Xue C., Ning L., He Y., Li Z., Xu X., He B. (2025). Nature.

[cit100] Li D., Zhao C., Zhang X., Zhao X., Huang H., Li H., Li F., Yuan J. (2025). Adv. Mater..

[cit101] Wang R., Zhang Y., Li Z., Wu L., Chen J., Liu X., Hu H., Ding H., Cao S., Wei Q., Wang X. (2025). J. Energy Chem..

[cit102] Liu L., Najar A., Wang K., Du M., (Frank) Liu S. (2022). Adv. Sci..

[cit103] Chen J., Ye L., Wu T., Hua Y., Zhang X. (2024). Adv. Mater..

[cit104] Zheng D., Pauporté T. (2024). Adv. Funct. Mater..

[cit105] Min L., Sun H., Guo L., Zhou Y., Wang M., Cao F., Li L. (2024). Adv. Mater..

[cit106] Min L., Sun H., Guo L., Wang M., Cao F., Zhong J., Li L. (2024). Nat. Commun..

[cit107] Kim Y.-H., Park J., Kim S., Kim J. S., Xu H., Jeong S.-H., Hu B., Lee T.-W. (2022). Nat. Nanotechnol..

